# DescFold: A web server for protein fold recognition

**DOI:** 10.1186/1471-2105-10-416

**Published:** 2009-12-14

**Authors:** Ren-Xiang Yan, Jing-Na Si, Chuan Wang, Ziding Zhang

**Affiliations:** 1 State Key Laboratory of Agrobiotechnology, College of Biological Sciences, China Agricultural University, Beijing 100193, China; 2 Bioinformatics Center, College of Biological Sciences, China Agricultural University, Beijing 100193, China

## Abstract

**Background:**

Machine learning-based methods have been proven to be powerful in developing new fold recognition tools. In our previous work [Zhang, Kochhar and Grigorov (2005) *Protein Science*, **14**: 431-444], a machine learning-based method called DescFold was established by using Support Vector Machines (SVMs) to combine the following four descriptors: a profile-sequence-alignment-based descriptor using Psi-blast *e*-values and bit scores, a sequence-profile-alignment-based descriptor using Rps-blast *e*-values and bit scores, a descriptor based on secondary structure element alignment (SSEA), and a descriptor based on the occurrence of PROSITE functional motifs. In this work, we focus on the improvement of DescFold by incorporating more powerful descriptors and setting up a user-friendly web server.

**Results:**

In seeking more powerful descriptors, the profile-profile alignment score generated from the COMPASS algorithm was first considered as a new descriptor (i.e., PPA). When considering a profile-profile alignment between two proteins in the context of fold recognition, one protein is regarded as a template (i.e., its 3D structure is known). Instead of a sequence profile derived from a Psi-blast search, a structure-seeded profile for the template protein was generated by searching its structural neighbors with the assistance of the TM-align structural alignment algorithm. Moreover, the COMPASS algorithm was used again to derive a profile-structural-profile-alignment-based descriptor (i.e., PSPA). We trained and tested the new DescFold in a total of 1,835 highly diverse proteins extracted from the SCOP 1.73 version. When the PPA and PSPA descriptors were introduced, the new DescFold boosts the performance of fold recognition substantially. Using the SCOP_1.73_40% dataset as the fold library, the DescFold web server based on the trained SVM models was further constructed. To provide a large-scale test for the new DescFold, a stringent test set of 1,866 proteins were selected from the SCOP 1.75 version. At a less than 5% false positive rate control, the new DescFold is able to correctly recognize structural homologs at the fold level for nearly 46% test proteins. Additionally, we also benchmarked the DescFold method against several well-established fold recognition algorithms through the LiveBench targets and Lindahl dataset.

**Conclusions:**

The new DescFold method was intensively benchmarked to have very competitive performance compared with some well-established fold recognition methods, suggesting that it can serve as a useful tool to assist in template-based protein structure prediction. The DescFold server is freely accessible at http://202.112.170.199/DescFold/index.html.

## Background

Template-based protein structure prediction methods (often known as comparative modeling and fold recognition) typically involve the following three steps. First, a (remote) homologous protein with known structure is identified as a template for a query sequence. The second step is to obtain an optimal alignment between the query sequence and the template sequence. Finally, a refined 3D model of the query protein can be generated based on the template structure. With more and more protein structural templates deposited in the current PDB database http://www.rcsb.org/pdb/home/home.do, template-based methods are increasingly powerful and their applications to many aspects of life sciences are widely explored [1].

The key step in template-based methods is to identify a structure template that shares a similar 3D structure with the query sequence. When the query protein shares significant sequence similarity with the template, classical sequence alignment methods, such as Blast [2], FASTA [3], Smith-Waterman [4] or Needleman-Wunsch [5] dynamic programming algorithm, are suitable and accurate in detecting their homologous relationship. Generally, the template-based method for dealing with such "easy" templates is referred to as comparative modeling. However, proteins with weak sequence similarity are also frequently found to share similar 3D folds. Such remote homology relationships can be hard to detect with classical sequence alignment methods. To find a template that shares only remote homology with the query protein, some profile-sequence (or sequence-profile) alignment methods like Psi-blast [6], Rps-blast [6], Impala [7], and Hidden Markov Models (HMM) [8] have been used, and they often result in a marked improvement. Nevertheless, the profile-sequence (or sequence-profile) alignment methods also perform poorly when the investigated protein pairs are situated in the twilight or midnight zone [9]. A lot of efforts have therefore been deployed to develop more sensitive and powerful remote homology detection techniques, called fold recognition. During the last decade, fold recognition has received considerable attention and a variety of elegant fold recognition methods (e.g., FFAS [10], 3D-PSSM [11], Fugue [12], mGenThreader [13], ORFeus [14], MUSTER [15], and SP5 [16]) have been developed. The overall good performance of these techniques has been widely demonstrated in the CASP [17] and CAFASP [18] competitions as well as in real-time LiveBench experiments [19].

The basic strategy of fold recognition methods consists in comparing the query sequence with all the structures within a fold library. According to the measured compatibility between sequence and structure, the fold recognition method can identify the template with the best fit. The well-established fold recognition methods can be roughly grouped into three main categories: (1) structure-seeded profile-based; (2) profile-profile alignment-based; and (3) machine learning methods-based. In the first category, 3D-PSSM and Fugue are probably the two best-known representative algorithms. For instance, 3D-PSSM is based on a hybrid fold recognition approach using sequence profiles and structure-seeded profiles (i.e., 3D profiles) coupled with predicted secondary structure information and solvation potential [11]. Grouped into the second category, the profile-profile alignment methods have recently been proven to be very powerful in remote homology identification as well as in generating accurate sequence alignments [20,21]. Generally, the profile-profile alignment method uses dynamic programming to obtain a direct alignment between two sequence profiles through Psi-blast searching [22,23]. To improve the performance of the profile-profile alignment, the structural information (e.g., predicted secondary structural information) was also frequently added to measure the similarity of two positional vectors [14,16]. In the third category, machine learning-based methods were employed to combine different sequence and structural information into fold recognition systems [13,24-27]. In mGenThreader [13], for instance, a neural network was used to combine pair-wise potentials, solvation potentials, and various alignment parameters. In the past several years, Support Vector Machines (SVMs) have also been widely used to build binary classifiers, which can allow the prediction of whether a sequence belongs to a single structural fold or not. Provided there are sufficient data in different protein folds, a set of binary classifiers can be trained and integrated into a fold recognition system (i.e. a multi-class predictor). A key step to establish an SVM classifier is to find effective kernel functions, which measure the similarity between any pair of protein sequences. There are some established kernel functions such as spectral kernel [28], profile-based string kernel [29], and mismatch string kernel [30].

A machine learning-based fold recognition method called DescFold was developed in our previous work [24]. In DescFold, any measurement between two proteins or any feature vector extracted from a protein sequence can be defined as a descriptor. For example, the amino acid composition of a protein can be regarded as a descriptor; the *e-*value obtained from a Blast search of protein *A *against protein *B *can also be considered as a descriptor between *A *and *B*. Based on such a broad definition, thirteen descriptors' fold identification capabilities were evaluated and four optimal descriptors were selected to construct the original version of DescFold with the assistance of SVMs. Although SVMs were frequently used to build discriminative models between various protein folds [27], it should be emphasized that the SVMs here were employed to distinguish structurally similar and dissimilar protein pairs. The four implemented descriptors were a profile-sequence-alignment-based descriptor using Psi-blast *e*-values and bit scores, a sequence-profile-alignment-based descriptor using Rps-blast *e*-values and bit scores, a descriptor based on the alignment of secondary structural elements (SSEA), and a descriptor based on the occurrence of PROSITE functional motifs [31]. Although the original DescFold was reported to significantly outperform a standard Psi-blast search, it showed weaker performance than some well-established methods when tested on the LiveBench-8 targets [24].

In the present study, we focus on developing an improved DescFold method through the following efforts. First, a profile-profile-alignment-based (PPA) descriptor was incorporated into the new DescFold method. Of the existing profile-profile alignment algorithms, COMPASS is one of the best-performing methods, and possess good computational efficiency [23]. Additionally, COMPASS is freely accessible to the community. In this work, the alignment scores resulting from the COMPASS algorithm [23,32] were defined as a PPA descriptor between a sequence pair. In the context of fold recognition, one of the aligned two sequences is regarded as a template, meaning that a structure-seeded profile is available for the template, which may contain different evolutionary information than a sequence profile derived from its homologous sequences. Moreover, the structure-seeded profile for the template sequence was generated by searching its structural neighbors with the assistance of TM-align [33]. Again, the COMPASS algorithm was further used to derive a profile-structural-profile-alignment-based descriptor (i.e., PSPA). Finally, we also set up a user-friendly web server for DescFold, and have made it freely accessible to the research community. Here, we present details on the improvement resulting from two newly introduced profile-profile alignment related descriptors, the construction of the DescFold web server, and the intensive benchmark results of testing DescFold against some state-of-the-art fold recognition methods.

## Results and Discussion

### The performance of individual descriptors based on the SCOP_1.73_1835 dataset

Based on the SCOP 1.73 version [34], we compiled a total of 1,835 sequence-dissimilar but structurally related proteins into a highly diverse protein dataset named SCOP_1.73_1835. Then, we used the SCOP_1.73_1835 dataset to benchmark the six different descriptor types in leave-one-out fold identification experiments. Each time, a protein in SCOP_1.73_1835 was selected as a "test" protein and the remaining proteins were regarded as a fold library. By calculating the pair-wise similarity scores defined in different descriptors, the "test" protein was scanned against the fold library and the protein with the most significant similarity score (i.e., the top hit) was recorded. In case the top hit and the test protein belong to the same SCOP superfamily, a correct fold identification was assigned. When the above experiment is performed over all the SCOP_1.73_1835 proteins, a descriptor's performance can be simply quantified in terms of sensitivity by counting the number of proteins with correctly identified structural homologs. More details about the construction of the different types of descriptors and the compilation of the SCOP_1.73_1835 dataset are outlined in the Methods section.

The sensitivities of fold identification using different descriptors are listed in Table 1. Of the four descriptors used in the original DescFold, the performance of the Rps-blast- and Psi-blast-based descriptors yield a sensitivity of 37.49% and 36.84%, respectively. Predicted secondary structure has been proven to be useful in protein fold recognition/classification [35], which can be effectively encoded by the SSEA-based descriptor [13,24,36]. The SSEA-based descriptor allows a correct identification rate of 28.56%. The motif-based descriptor is only able to generate successful fold identification for approximately 20% of the total protein sequences. Generally, the performance ranking of these four descriptors is in good agreement with the results from our previous study, although the descriptors were evaluated over two different datasets.

By capturing evolutionary information about residue preferences at different sequence positions in two profiles, profile-profile alignment has been shown to be very powerful in fold identification. Compared with the aforementioned four descriptors, the two profile-profile alignment related descriptors achieve better performance, and both descriptors allow successful fold identification for more than 50% of the tested protein sequences. Comparatively, the PPA descriptor is more powerful, and it outperforms the PSPA descriptor by nearly two percentiles (Table 1). Regarding the PSPA descriptor, the profile for one protein is derived from structural alignment results, which may contain different evolutionary information than the sequence profile inferred from the Psi-blast search results. By further combining the PPA and PSPA descriptors into our DescFold system, it is hoped that the overall performance of DescFold will be considerably improved.

**Table 1:** Sensitivity of fold recognition based on individual descriptors.

Descriptors	Sensitivity
SSEA	524/1835 = 28.56%
Psi-blast	676/1835 = 36.84%
Rps-blast	688/1835 = 37.49%
Motif	360/1835 = 19.62%
PPA	1083/1835 = 59.02%
PSPA	1052/1835 = 57.33%

### The overall performance of DescFold based on the SCOP_1.73_1835 dataset

The same strategy we used to evaluate the individual descriptors was used to assess the performance of DescFold (Table 2). For the purpose of comparison, computational experiments based on a combination of different descriptors were conducted. As shown in Table 2, the original DescFold (i.e., the results based on the SSEA-, Psi-blast-, Rps-blast-, and motif-based descriptors) can result in a sensitivity of about 56%. Representing local sequence features of proteins, the motif-based descriptor is alignment independent, implying that it should be complementary to the other alignment related descriptors. This can be clearly demonstrated by a 4% lower sensitivity when the motif-based descriptor was removed from the original DescFold system.

**Table 2:** Sensitivity of DescFold using different descriptors^a^.

Descriptors included	Sensitivity
SSEA + Psi-blast + Rps-blast	937/1835 = 51.06%
SSEA + Psi-blast + Rps-blast + motif	1025/1835 = 55.86%
SSEA + Psi-blast + Rps-blast + motif + PPA	1248/1835 = 68.01%
SSEA + Psi-blast + Rps-blast + motif + PPA + PSPA	1322/1835 = 72.04%

^a^The evaluation reflects fold identification performance of all proteins in the SCOP_1.73_1835 dataset. For each protein, only the generated top hit was taken into account.

**Figure 1:**
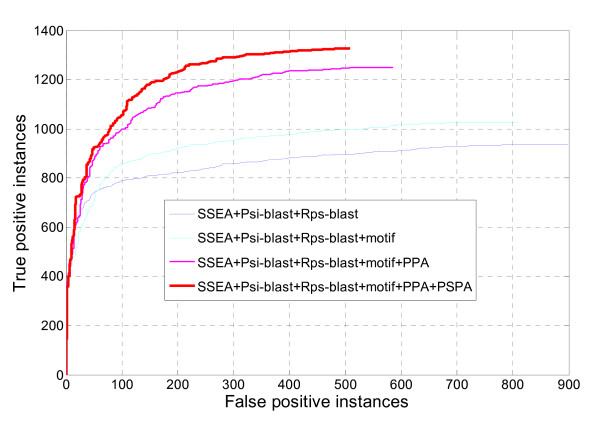
**Performance of fold recognition using different descriptors**. True positive instances versus false positive instances were used to examine the number of true positives out of 1,835 proteins identified by varying similarity scores.

As expected, the two profile-profile alignment related descriptors do provide considerable contributions to the new DescFold method, with has a nearly 16% higher sensitivity than that of the original DescFold. When the PSPA descriptor was not included in the DescFold system, a nearly 4% lower sensitivity was obtained, implying that the evolutionary information deposited in the two profile-profile alignment related descriptors are complementary to some extent. Moreover, a receiver operating characteristic (ROC) [37] curve, which plots true positive instances as a function of false positive instances for all possible thresholds, was also employed to measure the performance of the new version of DescFold. The improvement of DescFold resulting from the introduction of the PPA and PSPA descriptors is further revealed in the ROC curve (Figure 1). At a less than 5% false positive rate (i.e., 92 false positive instances) control, the new DescFold method is able to correctly recognize folds for 60.49% proteins, whereas only 46.16% proteins are successfully identified by the original DescFold method.

**Figure 2:**
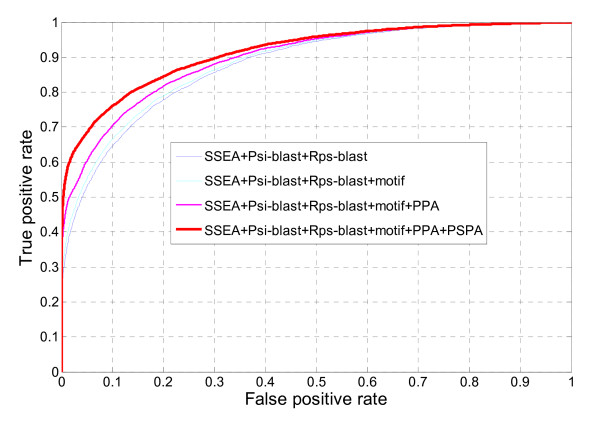
**Performance of remote homology identification using different descriptors**. True positive rates versus false positive rates were used to examine the number of true positives out of 8,244 protein pairs identified by varying similarity scores.

The above evaluation only reflects fold identification performance based on the generated top hits. As a more comprehensive evaluation, we also assessed DescFold's remote homology identification for all the protein pairs within the SCOP_1.73_1835 dataset via ROC analysis. As shown in Figure 2, the performance of DescFold when combining different descriptors has the same characteristics as the corresponding ROC curves in Figure 1. Additionally, the performance can be further quantified by the AUC and ROCn scores. The AUC score represents the corresponding area under the whole ROC curve, while the ROCn score is the area under the ROC curve up to the first n false positives. Since we pay more attention on the performance at low false positive rates, the ROCn score is more useful for practical applications. In addition to the AUC score, the ROC16,744, ROC83,720, and ROC167,440 scores (i.e., the ROCn values at 1%, 5%, and 10% false positive rates, respectively) are also listed in Table 3. At a less than 5% false positive rate control, the corresponding ROC83,720 score resulting from the new DescFold is approximately 0.008 higher than that of the original one (Table 3). Considering the corresponding sensitivity at this false positive rate control, the new DescFold is able to correctly identify approximately 69% of structurally similar protein pairs, providing an additional 15% improvement compared with the original DescFold (Table 3).

**Table 3:** The ROCn scores and the corresponding sensitivity values of DescFold using different descriptors.^a^

Descriptors included	ROC16,744 (Sn)^b, c^	ROC83,720 (Sn)^b, c^	ROC167,440 (Sn)^b, c^	AUC
SSEA + Psi-blast + Rps-blast	0.0029 (34.70%)	0.0209 (52.76%)	0.0506 (64.56%)	0.8768
SSEA + Psi-blast + Rps-blast + motif	0.0032 (37.73%)	0.0223 (55.25%)	0.0529 (66.49%)	0.8831
SSEA + Psi-blast + Rps-blast + motif + PPA	0.0041 (46.94%)	0.0256 (60.06%)	0.0584 (70.21%)	0.8962
SSEA + Psi-blast + Rps-blast + motif + PPA + PSPA	0.0050 (70.21%)	0.0305 (68.51%)	0.0668 (75.93%)	0.9143

^a^These measurements reflect the performance of remote homology identification for all protein pairs within the SCOP_1.73_1835 dataset.

^b^ROC16,744, ROC83,720, and ROC167,440 stand for the ROCn scores at 1%, 5%, and 10% false positive rates, respectively.

^c^The value inside the parentheses denotes the corresponding sensitivity.

**Figure 3:**
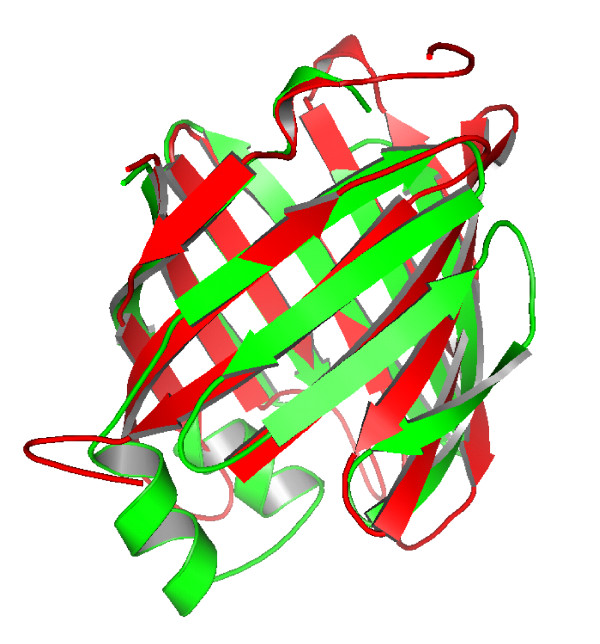
**Cartoon representation of two remote homologs (SCOP entries: d2a13a1 and d1hmsa_) successfully detected by DescFold**. The structural alignment between d2a13a1 (red) and d1hmsa_ (green) was carried out by using CE [51]. The RMSD for 121 structurally aligned residues is 3.6 Å, and the CE Z-Score is 5.2.

The performance of DescFold is further exemplified in the remote homology identification between two protein domains from the SCOP_1.73_1835 dataset (SCOP entries: d2al3a1 and d1hmsa_). Although d2a13a1 (a hypothetical protein from *Arabidopsis thaliana*) shares weak sequence similarity with d1hmsa (a muscle fatty acid binding protein from *Homo sapiens*), they are structural homologs (Figure 3) and belong to the same SCOP superfamily (lipocalins, SCOP superfamily index: b.60.1). When we searched d2a13a1 against the remaining 1,834 sequences using any individual descriptor, its superfamily partner (i.e., d1hmsa_) could not be ranked as the top hit. However, d1hmsa could be successfully assigned as the top hit when the search was carried out using our new DescFold.

### The DescFold web server and a large-scale benchmarking experiment on the SCOP_1.75_1866 dataset

**Figure 4:**
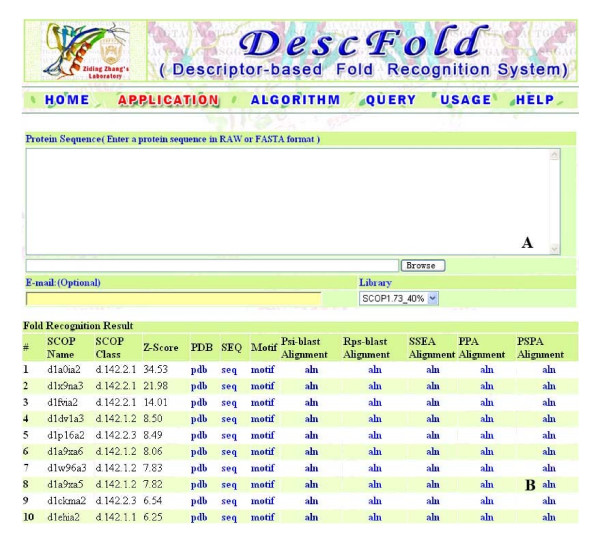
**Snapshot of the DescFold website**. (A) The submission page of DescFold. (B) The result page of DescFold.

Using the SCOP_1.73_40% dataset as the fold library, the DescFold web server was set up and is freely accessible at http://202.112.170.199/DescFold/index.html. Currently, a four-CPU DELL Linux machine with 16 GB of main memory hosts the DescFold web server. Generally, the computational time required for recognizing a protein's fold is reasonable for the current DescFold server; it takes about ten minutes to process a query sequence of 500 amino acids. Figure 4A is the submission page of the web server, and users can simply paste a protein sequence or upload a sequence file on this page to initiate the fold recognition process. When the recognition process is complete, users will be notified by e-mail. In the result page for fold recognition (Figure 4B), the top hits' Z-Scores, SCOP entries, sequence files and PDB files are listed. To quantitatively understand the reliability of the identified templates, we point out the confidence levels for different hits. Based on the current remote homology identification tests for all the protein pairs within the SCOP_1.73_1835 dataset (Figure 2), it was estimated that a Z-Score ≥ 10.0 yields a ≤ 1% error rate (i.e., 99% confidence level) and a Z-Score ≥ 6.0 indicates a ≤ 5% error rate (i.e., 95% confidence level). Moreover, the scores for all descriptors and sequence alignments generated from Psi-blast, Rps-blast, SSEA, PPA and PSPA are also listed in the result page, which can allow users to further judge the identified templates are correct or not. Among these sequence alignments, the PPA alignment based on the COMPASS algorithm is recommended to be used to obtain a 3D model for the query sequence with the assistance of some comparative modelling packages.

**Figure 5:**
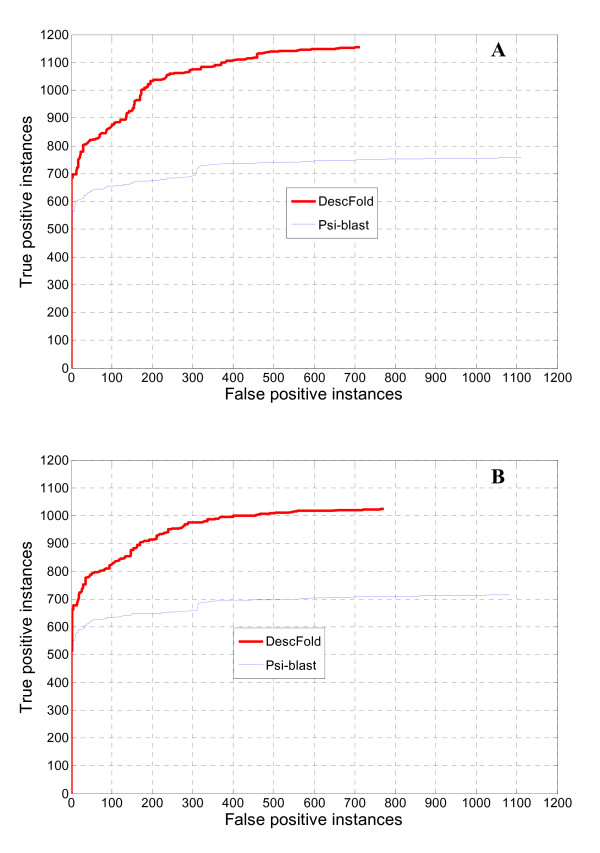
**Performance of DescFold based on the SCOP_1.75_1866 test set**. The performance was measured at the fold (A) and superfamily (B) levels, respectively.

By taking a similar strategy as reported in the literature of Auto-SCOP [38], we used a newer SCOP version (i.e., SCOP 1.75) to provide a large-scale benchmark for the current DescFold web server. 1,866 proteins were selected from SCOP 1.75 and compiled into a test dataset called SCOP_1.75_1866, which covers 171 different folds and 246 different superfamilies. All the 1,866 proteins share weak sequence similarities with the proteins in the fold library of DescFold (the Blast *e*-value for any protein pair between SCOP_1.75_1866 and SCOP_1.73_40% is > 0.1). On the other hand, 1,866 and 1,795 proteins in SCOP_1.75_1866 have at least one structural homolog in the fold library of DescFold at the fold and superfamily levels, respectively. Therefore, SCOP_1.75_1866 should be regarded as a good benchmarking dataset. More details about the selection of the SCOP_1.75_1866 dataset are outlined in the Methods section. We processed these 1,866 sequences via the current DescFold server and recorded the top hit for each sequence. According to the SCOP classification scheme, we measured the performance at the fold and superfamily levels. At the fold level, a correct prediction for a test protein can be assigned in case the top hit and the test protein belong to the same SCOP fold type. Generally, our DescFold is able to do the correct fold identification for 61.84% (i.e., 1,154/1,866 = 61.84%) test proteins, or some 21% more than a standard Psi-blast search. At a less than 5% false positive rate control, our DescFold method is able to correctly recognize folds for 46.25% proteins, whereas only 30.05% proteins are successfully identified by the standard Psi-blast search (Figure 5A). Note that the parameter settings of the standard Psi-blast search were the same as those used in deriving the Psi-blast-based descriptor. When assessing the performance at the superfamily level, a correct prediction means the top hit and the test protein should from the same SCOP superfamily. DescFold can correctly recognize structural homologs at the superfamily level for 57.05% (i.e., 1,024/1,795 = 57.05%) test proteins, which outperforms Psi-blast by a nearly 17% higher recognition rate. As further illustrated in the ROC curve (Figure 5B), DescFold also reveals a much better performance than the standard Psi-blast search.

### Comparison with some well-established fold recognition methods

In this work, our DescFold method was first benchmarked against some state-of-the-art fold recognition methods based on the LiveBench targets. As a real-time fold recognition benchmark program, every week the LiveBench server submits newly released PDB proteins to the participating fold-recognition servers, and evaluates the corresponding results. Here, we have selected the LiveBench-2008.1 targets (283 proteins) and LiveBench-2008.2 targets (513 proteins) as two reference test sets to compare the performance of DescFold and some well-established fold recognition methods. Although many fold recognition severs participated in the LiveBench-2008.1 and Livebench-2008.2 experiments, we compared our DescFold method with only five popular fold-recognition methods among them: 3D-PSSM [11], Fugue [12], mGenThreader [13], Inub [39] and FFAS [10].

Table 4 summarizes the performance of DescFold on the LiveBench-2008.1 targets, which is measured by the number of correct predictions with higher reliability than the 1-10 false prediction and the total number of correct predictions (i.e., sensitivity). Generally, the performance of DescFold is fully comparable to the five other fold recognition methods. Considering performance within ≤ 10 false positives, DescFold exhibits an overall higher fold identification rate than 3D-PSSM, a slightly better performance than Fugue and mGenThreader, and a lower identification rate than FFAS and Inub. Regarding the total number of correct predictions, DescFold is able to correctly identify fold types for 134 targets, which is also competitive to the five well-established methods. As defined by the developer of LiveBench, the targets can be divided into three categories: trivial, easy and hard targets. We separately list the corresponding sensitivity values on these three types of targets in Table 4. Generally, DescFold also shows reasonable performance in any category, although its relative rankings change slightly in three different categories.

**Table 4:** Comparison of receiver operator characteristics (< = 10 false positives) and sensitivity for different fold recognition methods based on all LiveBench-2008.1 targets.^a^

	Receiver operator characteristics (< = 10 false positives)^b^										Sensitivity^c^			
		
	1	2	3	4	5	6	7	8	9	10	All	Trivial	Easy	Hard
FFAS^d^	85	94	119	133	135	139	140	140	140	140	150	8	103	39
Inub^d^	73	89	106	116	120	121	121	121	121	121	134	6	91	37
Fugue^d^	61	79	81	85	87	96	101	102	104	104	135	8	95	32
mGenThreader^d^	77	89	89	90	90	93	97	97	98	98	143	8	97	38
3D-PSSM^d^	48	55	72	75	78	80	86	86	87	89	102	5	75	22
DescFold^e^	87	89	99	103	104	108	111	114	115	116	134	8	92	34

^a ^LiveBench-2008.1 contains 283 targets, which can be divided into 9 trivial, 109 easy and 165 hard targets. As defined by the developer of LiveBench, trivial targets means those proteins sharing strong sequence similarity to the other previously known structures, as measured by Blast using an *e*-value < 0.001. The division of easy and hard targets is based on whether a structural template can be identified by Psi-blast with an *e*-value < 0.001.

^b^1-10: number of correct predictions with higher reliability than the 1-10 false prediction.

^c ^Number of correct predictions for all, trivial, easy and hard targets, respectively.

^d^The results for FFAS, Inub, Fugue, mGenThreader, and 3D-PSSM were cited from http://meta.bioinfo.pl/results.pl?comp_name=livebench-2008.1

^e^The performance was evaluated based on the number of correctly assigned folds. We considered two hits as similar, provided that the Z-Score obtained by applying the CE structural alignment algorithm [51] was > = 4.0.

The performance of DescFold on the LiveBench-2008.2 targets is also comparable to the five other fold recognition methods (Table 5). Considering performance within ≤ 10 false positives, FFAS is still the best performing method among the five other fold recognition methods, whereas 3D-PSSM is ranked as the worst one again. The performance of DescFold is between these two, which is close to the three other methods (Inub, Fugue and mGenThreader) (Table 5). In our previous work, the same benchmark experiment was carried out based on the LiveBench-8 targets. Taking Fugue as a reference method, our original DescFold was benchmarked to have a considerably lower fold identification rate [24]. However, the new DescFold shows fully competitive performance with Fugue in both LiveBench-2008.1 and LiveBench-2008.2 experiments, suggesting that a clear improvement to DescFold has been made in this work.

**Table 5:** Comparison of receiver operator characteristics (< = 10 false positives) for different fold recognition methods based on all LiveBench-2008.2 targets.^a^

	Receiver operator characteristics (< = 10 false positives)^b^										Sensitivity^c^			
		
	1	2	3	4	5	6	7	8	9	10	All	Trivial	Easy	Hard
FFAS^d^	121	174	205	218	228	263	267	269	278	278	302	15	218	69
Inub^d^	29	34	126	149	183	195	209	210	211	228	257	14	189	54
Fugue^d^	129	186	199	219	221	223	224	225	225	225	285	16	213	56
mGenThreader^d^	179	197	205	211	215	215	216	222	232	232	290	16	215	59
3D-PSSM^d^	25	75	83	97	127	140	175	176	178	179	220	12	181	27
DescFold^e^	158	190	190	211	215	212	215	220	224	224	294	15	210	69

^a ^LiveBench-2008.2 has a total number of 513 targets, including 16 trivial, 246 easy and 256 hard targets. Please refer to the footnote of Table 4 for the definitions of trivial, easy and hard targets.

^b^1-10: number of correct predictions with higher reliability than the 1-10 false prediction.

^c ^Number of correct predictions for all, trivial, easy and hard targets, respectively.

^d ^The results for FFAS, Inub, Fugue, mGenThreader, and 3D-PSSM were cited from http://meta.bioinfo.pl/results.pl?comp_name=livebench-2008.2

^e^The performance was evaluated based on the number of correctly assigned folds. We considered two hits as similar, provided that the *Z*-Score obtained by applying the CE structural alignment algorithm [51] was > = 4.0.

The Lindahl dataset [40] was also employed to further benchmark the performance of our DescFold method. Based on the SCOP database (version 1.39), the Lindahl dataset contains 976 proteins, in which the sequence identity for any protein pair is < 40%. In this dataset, 555, 434 and 321 sequences have at least one matching structural homolog at the family, superfamily and fold levels, respectively. Taking the same strategy and procedures as we used with the SCOP_1.73_1835 dataset to develop the DescFold method, we retrained the DescFold method based on the Lindahl dataset. By employing the same assessment procedure as reported in the literature [16,25,40], the top 1 and the top 5 matched templates for each query sequence were used to evaluate the sensitivity of recognition performance. Since the Lindahl dataset was based on an old version of SCOP, it may be quite subjective to benchmark different methods based on this dataset. Ideally, the sequence and structural information of these 976 proteins should not be included in deriving the DescFold prediction models. More stringently, the sequence and structural homologs of these 976 proteins should also not be used. In the present study, we used the SCOP database (version 1.73) to derive the PSPA and motif-based descriptors. For instance, the PSPA descriptor used the SCOP_1.73_40% dataset to construct the structure-seeded profile, which may inevitably contain structural homologs of these 976 proteins. Meanwhile, the motif-based descriptor relied on the SCOP_1.73_95% dataset to derive the motif-fold compatibility, which may also utilize some sequence homologs of these 976 proteins. To allow for a fair comparison, we designed two DescFold predictors. In the first predictor (DescFold_I), both the PSPA and motif-based descriptors were skipped. In the second predictor (DescFold_II), the PSPA descriptor was still not considered, while the motif-based descriptor was kept. To derive the motif-based descriptor, however, these 976 proteins' sequence homologs in the SCOP_1.73_95% database were filtered by a Blast *e*-value threshold of 0.01.

We compared the performance of our DescFold with eight other fold recognition methods, including the standard Psi-blast search, HHpred [41], FOLDpro [25], Sparks [42], SP3 [43], SP4 [44], SP5 [16] and Fugue [12]. The corresponding results for these eight methods were cited from Refs. [16]and [25]. Table 6 shows the sensitivities of DescFold and the other well-established methods at the family, superfamily and fold levels, for the top 1 and top 5 matched templates, respectively. Although the PSPA was not considered, the performance of DescFold (i.e., DescFold_II) is fully comparable with the other methods. For prediction at the family level, the performance of DescFold is very close to that of well-established methods. For prediction at the superfamily level, DescFold is the best-performing method. Regarding the top 1 prediction at the fold level, DescFold surpasses all the tested methods except SP5. It is also worth mentioning that our DescFold (i.e., DescFold_I) is still competitive even when both the PSPA and motif-based descriptors are discarded.

**Table 6:** The sensitivity of different methods on the Lindahl dataset at the family, superfamily, and fold levels.^a, b^

Method	Family level (%)		Superfamily level (%)		Fold level (%)	
	
	Top 1	Top 5	Top 1	Top 5	Top 1	Top 5
Psi-blast^c^	71.2	72.3	27.4	27.9	4.0	4.7
Fugue^c^	82.2	85.8	41.9	53.2	12.5	26.8
FOLDpro^c^	**85.0**	**89.9**	55.0	70.0	26.5	48.3
HHpred^d^	82.9	87.1	58.0	70.0	25.2	39.4
Sparks^d^	81.6	88.1	52.5	69.1	24.3	47.7
SP3^d^	81.6	86.8	55.3	67.7	28.7	47.4
SP4^d^	80.9	86.3	57.8	68.9	30.8	53.6
SP5^d^	82.4	87.6	59.8	70.0	**37.9**	58.7
DescFold_I^e^	80.7	88.5	57.8	69.1	24.9	55.8
DescFold_II^f^	81.1	88.5	**60.6**	**72.4**	32.4	**59.8**

^a ^The sensitivity is defined by the percentage of query proteins having at least one correct hit ranked first, or within the top 5.

^b^Values in bold are the best results.

^c^The results were cited from Ref. [25].

^d ^The results were cited from Ref. [16].

^e ^DescFold_I was based on the SSEA-, Psi-blast-, Rps-blast-, and PPA-based descriptors.

^f ^DescFold_II was based on the SSEA-, Psi-blast-, Rps-blast-, motif-, and PPA-based descriptors.

Although many efforts were taken to make sure that the above two benchmark experiments were intensive and strict, we are still not able to guarantee a fully unbiased assessment. Regarding the benchmark based on the LiveBench targets, the fold libraries are different for the assessed methods, which may have some effect on the performance of the corresponding methods. For the comparative analysis based on the Lindahl dataset, the performance of other methods was originally collected from different literature. In this case, the sequence databases used to generate the profiles are not the same, which may result in different performance to some extent. Meanwhile, some methods may already have been significantly updated since their benchmark performance on the Lindahl dataset was published. As pointed out by Cheng and Baldi [25], such benchmark experiments can only provide a rough assessment rather than a very precise measurement. Even so, both of the aforementioned two benchmark experiments conclude that the performance of DescFold is fully comparable to some well-established peer methods.

## Conclusions

In this work, we developed an improved DescFold method by combining two new profile-profile alignment related descriptors (i.e., the PPA and PSPA descriptors). Due to the fact that the profile-profile alignment is able to capture more evolutionary information which was missed in our original DescFold, the new DescFold leads to a much better performance. The new DescFold method was benchmarked against some other state-of-the-art fold recognition techniques by using the LiveBench targets and Lindahl dataset. Our DescFold method demonstrates competitive performance in comparison to the existing methods. To allow for practical applications, we have made it freely accessible to the community through a user-friendly web-server.

Concerning future development, the following two efforts should be taken to maintain DescFold as a competitive fold recognition system. Firstly, the fold library of DescFold should be regularly updated. To provide a more comprehensive fold library, those experimentally determined structures which are not included in the SCOP database should also be taken into account. Secondly, seeking new descriptors is still the most important direction for development of a better predictor. On the one hand, machine learning based-methods allow the incorporation of more descriptors into a fold recognition system, which may yield better performance. On the other hand, the introduced descriptors will inevitably increase the complexity of the prediction model and obscure the contribution of each individual descriptor. Therefore, a new descriptor candidate should be carefully assessed before its acceptance for inclusion in the future versions of DescFold. Thus, we expect such machine learning-based methods will not only result in a fold recognition system with higher accuracy, but also strengthen our fundamental understanding of the evolutionary relationship between protein sequence and structure.

## Methods

### Datasets

In this work, we heavily rely on the SCOP database (version 1.73) [45] to construct the DescFold method. The corresponding SCOP sequences and structural data were obtained from the ASTRAL website http://astral.berkeley.edu/. To train and test the DescFold prediction models, two SCOP protein sequence subsets filtered by a 10% cut-off for sequence identity and an *e*-value threshold of 0.01 were downloaded from the ASTRAL website separately. Then, only sequences occurred in both of the above subsets were further kept. We also excluded sequences that are too short (less than 60 amino acids). Moreover, only a representative protein was reserved for each SCOP family. Finally, 1,835 protein sequences were kept and compiled into a dataset, which we named SCOP_1.73_1835 [see Additional file 1]. To construct the fold library of the DescFold web server, the SCOP_1.73_40% database with a total of 9,282 proteins was downloaded, in which the sequence identity among the proteins is equal to or less than 40%. The SCOP_1.73_40% database was also used as the database to search for structural neighbors for each template. Additionally, we also used the SCOP_1.73_95% dataset to derive the motif-based descriptor, in which the sequence identity for any sequence pair is ≤ 95%. A total of 15,273 protein sequences in the current SCOP_1.73_95% dataset were downloaded.

To perform a large-scale benchmarking on our DescFold server, a stringent test set was selected from a newer SCOP version (i.e., SCOP 1.75) based on the following criteria. Firstly, all proteins existed in SCOP 1.75 but not in SCOP 1.73 were downloaded. Secondly, only proteins sharing the fold types already existed in SCOP 1.73 were retained. Thirdly, proteins sharing a Blast *e*-value less than 0.1 with any protein in the SCOP_1.73_40% library were further discarded. Finally, 1,866 proteins from the SCOP 1.75 version were compiled into a test dataset called SCOP_1.75_1866 [see Additional file 2].

The NCBI non-redundant (NR) sequence database was downloaded from ftp://ncbi.nlm.nih.gov/blast/ (November, 2008). The NR database was further clustered at a cut-off of 90% identity (global alignment mode) by using CD-hit [46] and the resulting NR90 database, containing 4,205,215 sequences, was used to perform the Psi-blast search. To derive the motif-based descriptor, the PROSITE database (release 20.9) [31], which contains 1,322 patterns and 720 profiles, was obtained from http://www.expasy.org/prosite/.

### Descriptors

#### Psi-blast-based descriptor

The Psi-blast-based descriptor for a sequence pair *A *and *B *was obtained through the following steps. First, sequence *A *was searched against the NR90 database by Psi-blast for three iterations to generate a profile (i.e., profile *A*). The *e*-value cut-off for recruiting sequences in the profile was set as 0.001. Second, a Psi-blast search was performed on profile *A *against sequence *B *for another round. The above Psi-blast search resulted in two parameters, the expected value *evalue*_*Psi*-*blast*_*(A, B) *and the bit score *Score*_*Psi*-*blast*_*(A, B)*. In this work, *evalue*_*Psi*-*blast*_*(A, B) *was further modified according to the following equation.

Thus, the Psi-blast-based descriptor (i.e., *evalue_mod*_*Psi*-*blast*_*(A, B) *and *Score *_*Psi*-*blast*_*(A, B)*), can be used to measure the sequence similarity between *A *and *B*.

#### Rps-blast-based descriptor

The Psi-blast search can be conducted in a reverse way via Rps-blast (i.e., profile *B *against sequence *A*). As we derived the Psi-blast-based descriptor, the Rps-blast-based descriptor also results in two parameters *evalue_mod*_*Rps*-*blast*_*(A, B) *and *Score*_*Rps*-*blast*_*(A, B)*.

#### SSEA-based descriptor

To derive the SSEA-based descriptor for two query sequences *A *and *B*, the following three steps were involved. First, the secondary structures of the two query sequences were predicted by PSIPRED [47]. Second, the predicted secondary structural string for each sequence was converted into secondary structure elements such that "H" represents a helix element, "E" denotes a strand element, and "C" stands for a coil element. Third, the two secondary structure elements were aligned using a dynamic programming algorithm [5] with a scoring scheme proposed by Przytycka et al. [48]. The resulting alignment score *SSEA(A, B)*, ranging from 0 to 1, was regarded as the SSEA-based descriptor. For more details about the SSEA-based descriptor, please refer to our previous work [24].

#### Motif-based descriptor

In this work, the PROSITE motif library was used to derive the motif-based descriptor. First, the motif-fold correlation [49] in the SCOP database (i.e., SCOP_1.73_95%) can be quantified by a log-odds score *S *defined as:

where *p*(*motif*) and *p(fold) *are the individual probabilities of finding a particular sequence motif and a particular fold in the SCOP database, and *p*(*fold*, *motif*) is the corresponding joint probability. Furthermore, the motif-based compatibility between a query sequence and given folds can be expressed as:

where *S(fold|motif) *was calculated from equation 2 and the summation was performed over all motifs found in the query sequence and fulfilling the following criteria:

where *C *is an adjustable parameter, with 0.1 being an optimized value in this work. For a query sequence, the potential fold (PF) should be identified as the fold where *S*_*motif*_(*fold *| *sequence*) achieves a maximum. Then, the motif-based descriptor between two sequences *A *and *B *is defined as:

#### Profile-profile-alignment-based (PPA) descriptor

The COMPASS algorithm [23,32] was employed to derive a profile-profile-alignment-based descriptor between proteins *A *and *B*. First, a Psi-blast search was carried out to generate sequence profiles *A *and *B*, with the same parameter settings as we used to calculate the Psi-blast-based descriptor. Second, the two multiple alignments generated from the Psi-blast search (i.e., profiles *A *and *B*) were processed by COMPASS to obtain a profile-profile alignment. The resulting two parameters, *evalue*_*PPA*_*(A, B) *and *Score*_*PPA*_*(A, B) *were regarded as the similarity measurement between *A *and *B *(i.e., the PPA descriptor). Similar to Eq.(1), the *evalue*_*PPA*_*(A, B) *was further converted into *evalue_mod*_*PPA*_*(A, B)*.

#### Profile-structural-profile-alignment-based (PSPA) descriptor

Considering a protein pair *A *and *B *in the context of fold recognition, protein A is regarded as the query sequence and protein *B *is a structural template. Thus, the profile for protein *B *can also be obtained by searching its structural neighbours. To derive a PSPA descriptor between *A *and *B*, sequence profile *A *and structure-seeded profile *B *were generated. Sequence profile *A *was generated as described in deriving the Psi-blast-based descriptor, while the structure-seeded profile was obtained through the following steps. First, we searched structural template *B *against the SCOP_1.73_40% structural database using the TM-align structural alignment method [33] with default parameters. The search resulted in 9282 pair-wise structural alignments. Second, only those structural hits with a TM-align score > 0.6 were kept. Generally, a structural hit with a TM-align score > 0.6 is considered significant, meaning protein *B *and the corresponding hit share significant structural similarity. Moreover, we took sequence *B *as the reference sequence and no gaps were allowed, while we trimmed the structural hits' residues if they were aligned with the gap regions of sequence *B *in the corresponding pair-wise alignment. Finally, the corresponding pair-wise sequence alignments were combined into a multiple sequence alignment (i.e., structure-seeded profile *B*). When sequence profile *A *and structure-seeded profile *B *were prepared, the COMPASS algorithm was used again to derive the PSPA descriptor (*evalue_mod*_*PSPA*_*(A, B) *and *Score*_*PSPA*_*(A, B)*).

### Construction of DescFold

#### SVM learning

Based on the same strategy as detailed in our previous work, the aforementioned descriptors were combined into a fold recognition system termed DescFold with the assistance of SVMs. Similar to a 5-fold cross-validation, the protein pairs in the SCOP_1.73_1835 dataset (i.e., 1835 × 1834/2 = 1,682,695 pairs) were divided into five subsets of nearly equal size. Here, the SVM was trained to distinguish two different types of protein pairs (i.e., structurally similar and structurally dissimilar pairs). For the first type of protein pairs (i.e., positive instances), both proteins belong to the same superfamily. For the second type of protein pairs (i.e., negative instances), the two proteins are from different superfamilies. Of the total 1,682,695 protein pairs, 8,244 pairs were considered positive instances and their labels were set to + 1, while1,674,451 pairs were considered negative instances and their labels were set to -1. The aforementioned six descriptors were input as the feature vector for each protein pair, which contains a total of ten parameters. Taking a protein pair *A *and *B *as an example, the corresponding ten parameters are *evalue_mod*_*Psi*-*blast*_*(A, B)*, *Score*_*Psi*-*blast*_*(A, B)*, *evalue_mod*_*Rps*-*blast*_*(A, B)*, *Score*_*Rps*-*blast*_*(A, B)*, *SSEA(A, B), Motif_Score(A, B)*, *evalue_mod*_*PPA*_*(A, B)*, *Score*_*PPA*_*(A, B)*, *evalue_mod*_*PSPA*_*(A, B)*, and *Score*_*PSPA*_*(A, B)*.

To predict whether a given protein pair were structurally similar or dissimilar, the subset to which this pair belongs was labeled the "test" set, whereas the four remaining subsets were labeled "training" sets. SVM models were developed for each of the "training" sets. The ratio of the positive to negative instances in each training dataset is approximately 1:200. An unbalanced training dataset will affect the prediction performance of the established SVM models and we found that the optimal ratio in the training set was 1:2.5. Each training dataset was adjusted by discarding a random selection of the negative pairs prior to training. The whole training resulted in four separate SVM models, the prediction score being obtained as an average value over the decision values from the four different SVM models. Furthermore, the raw prediction score (RPS) was further converted into a Z-Score. We randomly selected 3000 pairs from the 1,682,695 protein pairs, and calculated the average value (AVE) and standard deviation (SD) of these pairs' prediction scores. For a query sequence, a Z-Score can then be calculated: *Z *= (*RPS *- *AVE*)/*SD*.

Libsvm [50] was employed as the SVM algorithm in our work. The applied kernel was the linear function and the other parameters were set to their default values. We also tried the automatic parameter optimization provided by Libsvm, but it did not result in a better performance. Instead of performing any further parameter optimization, we only used the default SVM parameters in our DescFold method. According to the randomized grouping of five subsets, the 5-fold cross-validation was repeated 5 times. Finally, the average performance was reported.

Of the ten input features (parameters) used in building the SVM models, it is interesting to quantify the relative importance of each feature in classifying structurally similar and dissimilar protein pairs. The feature selection tool fselect.py http://www.csie.ntu.edu.tw/~cjlin/libsvmtools/#6 provided by the Libsvm developer was employed to measure the relative importance of each feature. For each feature, an F-score can be calculated from fselect.py. Generally, the larger the F-score is, the more important this feature is. As shown in Table 7, *Score*_*PSPA*_*(A, B) *tends to be the most important, while *Motif_Score(A, B) *is ranked as the weakest feature.

**Table 7:** The F-scores of ten input features used in building the SVM models.

Feature	F-Score
*Score*_*PSPA*_(*A*, *B*)	0.421
*SSEA(A, B)*	0.368
*Score*_*PPA*_(*A*, *B*)	0.279
*evalue_mod*_*PSPA*_(*A*, *B*)	0.217
*evalue_mod*_*PPA*_(*A*, *B*)	0.162
*Score*_*Psi*-*blast*_(*A*, *B*)	0.135
*Score*_*Rps*-*blast*_(*A*, *B*)	0.119
*evalue_mod*_*Psi*-*blast*_(*A*, *B*)	0.081
*evalue_mod*_*Rps*-*blast*_(*A*, *B*)	0.062
*Motif_Score(A, B)*	0.026

#### Construction of the web server of DescFold

To aid the research community, a web server for DescFold was constructed and is freely available at http://202.112.170.199/DescFold/index.html. To sufficiently represent the known protein structural space, the 9,282 proteins in the SCOP_1.73_40% dataset were used as the fold library. For computational efficiency, the Psi-blast-derived profiles, predicted secondary structure elements, *S*_*motif*_(*fold*|*sequence*), and structure-seeded profiles of the template proteins were pre-calculated. To search a query sequence against the fold library (i.e., SCOP_1.73_40%), a total of 9,282 protein pairs were involved. For each protein pair, the corresponding six descriptors were calculated. Then, the resulting ten parameters were used as the input for five SVM models trained in the above section, and the prediction score was obtained as an average value over the decision scores from the five different SVM models. Moreover, the prediction scores for all protein pairs were converted into Z-Scores. Finally, the top hits ranked by Z-Scores were reported. Users have options to display the top hits by setting the number of hits and the cut-off of Z-Score. The default number is ten and the maximal number is 50.

## Availability and requirements

**Project Name**: DescFold

**Project home page**: http://202.112.170.199/DescFold/index.html

**Operating system**: Online service is web based; local version of the software should be run on a Linux platform.

**Programming language**: Perl.

**Other requirements**: None.

**License**: Free.

**Any restrictions to use by non-academics**: None.

## Authors' contributions

RXY wrote codes, developed the web server, and drafted the manuscript. JNS and CW participated in the method assessment. ZZ directed the research and critically revised the manuscript. All authors read and approved the final manuscript.

## Supplementary Material

Additional file 1 **Contains the primary sequences of the SCOP_1.73_1835 dataset (SCOP_1.73_1835.txt). **All the sequences are provided with FASTA format. Click here for file

Additional file 2 **Contains the primary sequences of the SCOP_1.75_1866 dataset (SCOP_1.75_1866.txt). **All the sequences are provided with FASTA format. Click here for file
